# Supplementary material for: Are carbon emissions trading and green financial instruments synergistic? -Comprehensive quantitative research based on content analysis
Source: PLoS One. 2024 Mar 7;19(3):e0298601. doi: 10.1371/journal.pone.0298601 (PMC10919601; doi:10.1371/journal.pone.0298601)
Supplement: S1 Data — (ZIP) [file pone.0298601.s001.zip › Data/地级市Z_F工作报告.zip!/║╙─╧/╚2├┼╧┐╩╨/└┤╫╘╣1⁄2╓┌║┼í╛┬φ┐╦╩2╛▌═°í┐_2008.docx]

各位代表：

　　现在，我代表市人民政府，向大会作政府工作报告，请予审议，并请各位政协委员提出意见。

一、2007年工作回顾

　　过去的一年，市政府在省委、省政府和市委的正确领导下，在市人大、市政协的监督支持下，团结和带领全市人民，紧紧围绕再造一个三门峡、跻身中原崛起第一梯队的奋斗目标，深入贯彻落实科学发展观，加快构建和谐社会，圆满完成了市五届人大一次会议确定的各项任务。

　　（一）综合实力不断增强，质量效益进一步提高。全年生产总值突破500亿元大关，完成518.56亿元，比上年增长18.7%；人均GDP达到2.3万元。地方财政一般预算收入完成29.36亿元，增长35.7%，收入规模和增速分别居全省第九位和第二位。规模以上工业企业完成增加值265.24亿元，增长28.1%；实现利润115.73亿元，增长61.5%。高新技术产业完成增加值17.98亿元，增长19.7%。万元GDP能耗下降5.05%，化学需氧量和二氧化硫排放量分别下降14.9%和10.1%。

　　（二）项目建设硕果累累，发展后劲持续增强。全社会固定资产投资完成291.4亿元，比上年增长27.2%。列入市委、市政府督查范围的116个工业重点项目，累计完成投资104.2亿元。电网建设成效显著；开曼氧化铝三期、新源燃料14万吨甲醇等50多个项目建成投产；新增生产能力煤炭100万吨、氧化铝80万吨、黄金3万两、浓缩果汁5万吨。三门峡火电厂三期、义马1000万方煤气等项目前期工作积极推进。建成各类工业项目聚集区15个，入驻项目150个，其中投资3000万元以上项目128个。

　　（三）农村经济健康发展，新农村建设稳步推进。特色产业进一步壮 大，果品总产量达到13.5亿公斤，“岭宝苹果”、“陕州红梨”等成为具有较大影响力的知名品牌。狠抓畜牧产业化养殖，规模场户饲养占全市总量的41%，肉类总产量7.8万吨、禽蛋5万吨、牛奶2.3万吨，分别增长5.4%、9%、25%。林业生态县、乡、村三级联创稳步推进，林业产业初具规模。农业龙头企业快速发展，销售收入超百万元的企业达到100家，13家进入省级龙头企业行列。农业标准化水平不断提高，63个无公害农产品基地通过省级认定。农业信息化工作被树为“中国农业信息化领先地区”和“全国新农村建设信息化示范市”。新农村建设各项工作扎实推进，50个示范村都有支柱产业。实现劳务收入15.1亿元，完成搬迁扶贫651户、2677人。

　　（四）城市基础设施不断完善，城镇化水平明显提升。涧河五期治理主体完工，茅津北路、甘棠南路、上官南路等道路建成通车，涧南城区改造进展顺利。城市引水工程主体完工，集中供气和城区绿化、亮化等工程稳步实施。“城中村”改造工程正式启动。市区生活垃圾处理场扩建工程完工，医疗垃圾处理场基本建成，县（市）污水处理厂全部投入运行。“四城联创”实现年度工作目标。副中心城市基础设施建设稳步推进，小城镇有序发展，全市城镇化率达到42.4%。

　　（五）各项改革逐步深入，外向型经济迈上新台阶。财税、金融、流通、投资等一些重点领域和关键环节的改革取得突破。直接利用外资1.87亿美元，比上年增长35.3%；引进市外资金60亿元，增长25%。成功举办第十三届三门峡国际黄河旅游节暨投资贸易洽谈会，积极参加中博会，签约总金额389.1亿元，创历史最高水平。外贸出口稳定增长，出口创汇完成1.77亿美元，增长16%。规模以上非公有制工业企业完成增加值106.35亿元，增长30.4%，对工业增长的贡献率达到42.1%。社会消费品零售总额117.27亿元，增长17.8%；居民消费价格总指数为106%。

　　（六）人民生活明显改善，社会事业全面发展。城镇居民人均可支配收入突破万元大关，增长18.1%；农民人均纯收入4033元，增长17.4%。市委、市政府年初确定的“十件实事”顺利完成。新建和改造农村公路1034公里，基本实现“村村通”目标。解决了15.4万农村人口的安全饮水问题；新增农村沼气用户2万户；农村“无电户”全部通电。落实“两免一补”资金7906万元，资助贫困学生近55.2万人次；职业教育得到发展，高、中等职业院校在校生分别达到8542人和3.2万人。乡镇卫生院改造全部完成；新型农村合作医疗覆盖全市。新增城镇就业5.63万人，城镇登记失业率为3.31%。养老、失业等五项社会保险费征缴额6.84亿元。农村居民最低生活保障制度全面建立，城市低保实现了动态管理下的应保尽保；救灾工作扎实有效，实现了省委、省政府提出的“六个确保、两个恢复”的目标；国有煤矿棚户区改造竣工50万平方米，超额完成省定目标。经济适用房竣工10.43万平方米，廉租房制度基本建立。广播电视“村村通”工程有序推进。文化事业繁荣发展，灵宝西坡考古项目入选“年度全国十大考古新发现”，《虢都遗恨》荣获国家舞台艺术精品工程提名奖，虢国澄泥砚、灵宝皮影、卢氏根雕等荣获“河南省知名文化产品”，豫西文化长廊建设进展顺利。全民健身活动蓬勃开展，竞技体育水平不断提高。

　　（七）应急管理体系不断完善，行政效能和服务水平逐步提高。全面贯彻《突发事件应对法》，市、县两级应急管理组织全部建立，制定完善各类应急预案84个，妥善处置各类突发事件30余起。陕县“7·29”淹井事件救援成功，69名被困矿工全部获救，创造了我国煤矿救援史上的奇迹；卢氏县特大洪涝灾害抢险救援取得胜利，灾民生产生活得到妥善安置。修订完善政府工作制度与规范，不断促进政府管理规范化；行政服务中心“窗口”功能得到加强，服务效率和水平明显提升；大力推进电子政务，应急指挥网络建设走在全省前列。

　　（八）精神文明建设扎实推进，民主法制建设全面加强。深入开展“讲正气、树新风、谋发展、促和谐”主题教育活动，精神文明创建活动不断深化，“双创”和“两抓一促”取得实效，涌现出牛江涛、郑秀珍等一批先进典型。自觉接受市人大及其常委会和市政协监督，办理人大代表建议119件、政协委员提案333件，满意和基本满意率分别达到100%和99.7%。坚持依法行政，政府法制工作不断加强。全面落实安全生产责任制，安全生产形势稳定好转。创新群众工作模式，加强社会治安综合治理，平安三门峡建设成效显著。双拥工作不断加强，军政、军民关系进一步巩固，实现“全国双拥模范城”三连冠。认真落实党风廉政建设责任制，廉政建设和反腐败斗争取得新成效。审计、外侨、对台、老龄、档案、史志、人防、无线电、民族宗教、妇女儿童、河务移民、残疾人救助等各项事业取得新进展。

　　这些成绩，是在国家宏观调控政策趋紧、遭遇百年不遇洪涝灾害和经济连续高速增长的形势下取得的，确属来之不易。值此，我代表市人民政府，向全市各行各业的广大工人、农民、知识分子、干部、人民解放军指战员、武警官兵以及社会各界人士致以崇高的敬意和衷心的感谢！

　　同时，我们也清醒地看到，经济社会发展的过程中还存在一些困难和问题：经济增长比较粗放，技术创新能力不强，第三产业发展滞后，转变发展方式任务繁重；资金、土地、环境等约束加剧，发展制约性因素增多；产业结构调整任务艰巨，能源、原材料工业比重较大，节能减排形势严峻；农业基础依然薄弱，持续增收难度加大；部分群众生产生活还比较困难，关系群众切身利益的上学就医、劳动就业、社会保障等方面还存在不少问题；个别地方和部门依法行政意识不强；一些干部还存在官僚主义、形式主义等现象。对此，我们一定高度重视，并在今后的工作中认真加以解决。

二、2008年政府工作的总体要求和主要预期目标

　　今年政府工作的总体要求是：以党的十七大精神为指导，深入贯彻落实科学发展观，紧紧围绕再造一个三门峡、跻身中原崛起第一梯队的奋斗目标，进一步突出优化经济结构和转变发展方式这一主线，更加注重质量效益，更加注重经济运行的稳定性，更加注重经济社会发展和生态建设的一致性，更加注重改革发展稳定的协调性，推动国民经济又好又快发展，努力使三门峡在新的起点上实现更大跨越。

　　主要预期目标是：全市生产总值增长13%；工业增加值增长14.5%；全社会固定资产投资增长19%，其中城镇固定资产投资增长20%；地方财政一般预算收入增长15%；社会消费品零售总额增长15%；实际直接利用外资增长25%，进出口总额增长16%；城镇居民人均可支配收入增长9%，农民人均纯收入增长7%。城镇登记失业率控制在4.5%以内；人口自然增长率控制在6.5‰以下；单位生产总值能耗下降5.6%，化学需氧量和二氧化硫排放量分别控制在1.87万吨和15.33万吨以内。

三、切实转变经济发展方式

　　（一）大力推进产业结构优化升级。调整优化工业结构。加快五大基地建设，拉长产业链条，扩大产业集群，壮大支柱产业。能源工业，抓住国家“上大压小”的政策机遇，做好100万千瓦火电机组容量置换项目的前期工作；强力推进风力发电项目。铝工业，集中精力抓好氧化铝的就地转化和电解铝的深加工，重点推进高终端铝制品开发建设，加快铝合金轮毂扩建、铝铸件、铝箔等项目进度，建设与氧化铝产能相适应的铝加工能力。煤化工，抓住国家支持义马煤化工产业的有利机遇，抓好煤气、甲醇等项目建设，确保煤化工产能得到大幅提升。有色金属综合利用及深加工，继续推进黄金深部探矿，进一步提高金、银、铅、铜等伴生矿的综合开发和利用水平。林果业生产加工，加快果品加工行业资源整合，提升果品深加工的规模和水平，引导产业有序竞争，保持和扩大浓缩果汁产能全国领先优势。大力培育接续产业。加快高新技术产业项目发展，在进一步扶持壮大铝合金轮毂、生物酶制剂、液晶技术等新兴企业的基础上，在新材料、生物工程、医药、信息与现代制造等领域，抓紧实施一批具有比较优势的高新技术型项目，力争实现新突破。着手制定接续产业发展规划，重点发展机械制造、农副产品加工、生物制药等产业，强力开发可再生能源，做大相关接续产业。积极推进企业战略重组。强力推进电解铝与氧化铝企业、大型煤炭与化工企业以及水泥行业、果汁行业的整合，培育产业链条完整的大型骨干企业集团，力争更多的企业进入省百户重点工业企业和50户高成长性工业企业行列。积极协调煤、电、油、运等生产要素，确保工业经济稳健运行。

　　（二）突出抓好重点项目建设。加快工业项目建设。确保天瑞集团20万吨铝合金铸件、锦荣公司180万吨干法水泥、金源公司硫铁化工综合利用二期、渑池4万吨高强度石油支撑剂等续建项目年内建成投产；力争东方希望100万吨离子膜烧碱和120万吨PVC管材、渑池3500吨果酒、义马20万吨醋酸等项目尽快开工建设；积极做好工业园3000吨多晶硅、义马1000万方煤气等重大项目的前期工作。加强基础设施建设。全面推进郑西铁路客运专线、运（城）三（门峡）高速公路黄河大桥、义煤集团采煤沉陷区综合治理和西气东输二线、兰州—长沙输油管道等一批重大基础设施项目建设，保证完成年度计划。加强供电基础设施建设，构建以500千伏为主的骨干供电网络。加速推进工业园的开发与建设。第一批入园的6个项目要尽快建成投产，2万件大型化工机械装备、1万吨氧化铝清洁生产等第二批入园项目要抓紧开工。抓好工业园基础设施建设，理顺管理机制，出台更优惠的政策，吸引更多的投资者。

　　（三）加快发展服务业。完善发展规划，构筑高增值、多层次、广就业的现代服务业体系。加快提升商贸、餐饮等传统服务业，大力发展金融保险、法律服务、会计审计、信息技术、社区服务等现代服务业。积极发展第三方物流，鼓励大中型工业企业分离物流业务、开展物流外包。搞好七海物流、九州通医药配送基地、三门峡西物流园区等项目建设。选择一批现代服务业优势企业给予重点扶持，争取进入省百户重点服务业企业盘子。抓好豫西大峡谷、仰韶大峡谷、卢氏双龙湾、函谷关等景区基础设施的配套建设，全力开发“文化山水”旅游品牌和相关产品，提升服务档次。大力实施豫晋陕旅游区域合作，推进无障碍旅游。推行建筑行业的整合重组，培育壮大骨干企业，力争年产值超5000万元的企业达6家以上。

四、统筹城乡协调发展

　　（一）加快发展现代农业。全面落实强农惠农政策。认真执行粮食直补、良种补贴、农机具购置、家电下乡等各项政策，确保补贴落实到位。继续推进“万村千乡”市场工程，实现2/3以上行政村有便民超市。大幅度增加农业农村投入，搞好农田水利基本建设和高效农业综合开发，稳定提高农业综合生产和抵御自然灾害能力。大力发展特色农业。围绕林果、畜牧、烟叶等优势主导产业，按照规模化生产、区域化布局的要求，加快特色农产品产业基地建设，构筑现代农业产业体系，力争特色农业产值占农业总产值的比重达到85%以上。新栽果树16万亩，稳定增加果品产量；大力发展生物质能源林、用材林、经济林等特色基地，不断发展壮大林业经济。抓好重大动物疫病防治，新建高标准养殖小区25个，总量达到140个；落实烟叶种植面积24万亩，稳步提高烟叶质量。加快农业“三化”进程。扎实开展重点农业龙头企业竞赛活动，产值超5000万元的企业达到15个以上。抓好景源、鑫源、缘分、康瑞等出口创汇型龙头企业发展，大力发展外向型农业。积极发展农民专业合作组织，力争每个乡（镇）至少有1家运作规范、市场竞争力强、辐射带动面广的农民专业合作社。深入开展农业标准化“五级联创”活动，加快建设一批高标准的优质农产品生产基地。严格农产品市场准入制度，提高农产品质量安全水平。深入推广农业信息化“三电一厅”模式，为农民提供更有效、更实用的服务。大力推广农业先进技术，农作物良种覆盖率达到90%以上，先进技术入户率达到80%以上。

　　（二）分类推进社会主义新农村建设。大力发展农村经济，努力增加农民收入。用3年时间，有效改善环境卫生状况和村容村貌，推动社会主义新农村建设取得较大进展。人均收入超过5000元的村庄，重点提高建设和管理水平，加快实施统一的垃圾处理、给排水等基础设施建设，加强生态绿化和环境美化，提高村务管理水平，推动有条件的村庄逐步发展成农村新社区，建成社会主义新农村样板。人均收入在3000—5000元的村庄，重点搞好村庄规划，硬化村中道路，搞好村庄周围、庭院和通道绿化；发展农村户用沼气，积极改灶、改圈、改厕，实行垃圾集中处理，有条件的要通自来水；力争使多数村庄有卫生室、有小超市、有文化大院、有健身活动场所，建设饮水安全、能源清洁、村庄绿化、街道明亮、环境整洁、文明和谐的新农村。人均收入在3000元以下的村庄，坚持与扶贫开发紧密结合，集中资金，创新机制，突出重点，抓好25个重点村的整村推进和搬迁扶贫，帮助群众脱贫致富。大力实施“阳光工程”、“雨露计划”和新型农民培训工程，全年培训农民25万人次。继续推进“沼气工程”建设，重点村沼气普及率达到60%以上。

　　（三）加速推进城镇化。加快中心城市建设。严格执行城市总体规划，认真编制控制性详细规划。开工建设郑西铁路三门峡新客车站，规划建设市区与新客车站连接通道。巩固涧河治理成果，提升城市重点景区的品位和档次。抓住西气东输二线建设机遇，提前做好天然气利用规划、城市配套管网工程等前期工作。搞好城市管理。创新管理理念、体制和手段，突出抓好市容环境、市政设施、园林绿化、公共交通等方面的精细化管理。全面推进“四城联创”，园林城市创建，要进一步提升涧南公园、陕州公园品位，努力打造亮点工程，为明年验收打好基础；卫生城市创建，要进一步加强市容环境建设，积极开展先进小区、先进单位创建活动；环保模范城市创建，要着力改善环境质量，突出抓好各项指标落实；文明城市创建，要大力推进“三和社区”建设，提高整体创建水平。加强城市规划监察执法力度，严厉打击城市违法占地、违法建设、私搭乱建等行为。加强节地、节水、节材工作，提高城市集约、节约发展水平。稳步推进“城中村”和旧城改造。坚持政府引导、统一规划、市场运作、群众自愿原则，拓宽融资渠道，加大改造力度，力争取得实质性进展。把“城中村”和旧城改造与建设特色街区、大力发展第三产业结合起来，进一步优化城区产业结构。吸引开发商参与县城改造与建设，把县（市）城区建成各具特色的区域性经济、文化中心。

　　（四）大力发展县域经济。义马—渑池城区要继续围绕培育壮大能源、煤化工和铝工业等主导产业，加快快速通道建设进度，全面推进城区的实质性对接；陕县、湖滨区要依托服务中心城市，加快发展农产品加工、高新技术产业、现代物流和服务业；灵宝市要充分发挥有色金属综合利用、林果业生产加工的优势，进一步提升辐射带动能力；卢氏县要深入挖掘生态旅游资源，搞好特色农副产品开发加工，培育比较优势明显、地方特色鲜明的支柱产业。深化城镇户籍制度改革，继续推进城乡一体化试点，鼓励建设城乡统一的基础设施和公共服务体系，促进城乡协调发展。完善工业聚集区功能，承接产业转移

五、积极推进以改善民生为重点的社会建设

　　（一）继续办好“十件实事”。在全面落实省委、省政府“十大实事”的基础上，结合我市实际，今年要集中力量办好关系人民群众切身利益的“十件实事”：一是城市引水工程实现全面通水。二是新建大岭路涧河立交桥，完成黄河路西段和虢国东路改造。三是启动台上区集中供热工程，改善市区供热质量。四是在市区新建1所小学和1所福利院。五是加快天鹅湖湿地公园建设。六是启动群众文体活动中心建设。七是全面开展城镇居民医疗保险，启动市中心医院病房大楼改建工程。八是实施市级广播电视节目“村村通”工程。九是大力推进“城中村”和旧城改造工程，城镇建设廉租住房1万平方米，农村搬迁扶贫3086人。十是切实改善农村生产、生活条件（改造县乡公路300公里、通村公路200公里；解决农村安全饮水6万人；新增农村户用沼气2万个；新建260个农村便民超市；新改建9所农村敬老院，农村五保对象集中供养率达到40%；免费实施白内障复明手术5000例）。

　　（二）优先发展教育事业。全面实施城乡免费义务教育，提高农村中小学公用经费标准，增加贫困家庭寄宿生生活费补助。免费向农村义务教育阶段学生提供教科书；从今年秋季起，免除城市义务教育学杂费。全面落实高、中等职业教育贫困生资助政策，认真解决农民工子女就学和城市部分中小学大班额问题。加大农村中小学危房和薄弱学校的改造力度，充实教学仪器设备，改善办学条件。进一步优化配置远程教育设施，推进优质教育资源共享。加快普及高中阶段教育，着力提高基础教育水平。稳步发展高等教育，大力发展职业教育，积极发展民办教育。加大教师培训力度，提高教师队伍素质，逐步改善教师待遇。

　　（三）大力发展医疗卫生事业。稳步实施医疗卫生体制改革，建设覆盖城乡居民的公共卫生、医疗服务、医疗保障、药品供应保障四大体系，为群众提供安全、有效、方便、廉价的医疗卫生服务。健全社区卫生服务机构财政补助机制，完成200所村级卫生室建设，积极开展农村甲级卫生室创建工作。规范医疗服务收费价格，扩大按病种付费试点，抓好重大传染病防治。扶持中医药事业发展，努力将市中医院建成国家重点市级中医院。异地新建三门峡市中心血站。

　　（四）加快发展文化事业和文化产业。推进豫西文化长廊重点区域建设，构建覆盖城乡的公共文化服务体系。充分发挥文化馆、图书馆和文化站的作用，加快发展公益性文化事业。进一步推动文化信息资源共享、城镇数字电影广场工作，实施农村电影放映工程。加大重点文物和非物质文化遗产保护、开发和利用，认真做好崤函古道申报世界文化遗产工作，确保仰韶村、庙底沟和西坡三大遗址保护规划通过国家验收。深入开展“中国楹联文化城市”创建活动，提高文化品位和内涵。强化文化市场监管，深入开展“扫黄打非”。继续实施“两抓一促”工程，深化精神文明创建活动。广泛开展科教、文体、法律、卫生进社区和科技、文化、卫生“三下乡”活动，丰富社会文化生活。

　　（五）认真做好就业再就业和收入分配工作。建立健全创业服务体系和平等就业制度，多渠道开发就业岗位，新增城镇就业4万人。加大再就业援助力度，帮助1.3万名下岗失业人员实现再就业，及时解决“零就业”家庭就业困难。做好大中专毕业生、退伍转业军人、残疾人就业工作。加强农民工技能培训，新增农村富余劳动力转移就业3万人。继续深化收入分配制度改革，建立职工工资正常增长和支付保障机制，逐步提高最低工资标准。认真落实《劳动合同法》，保护从业者合法权益，落实职工带薪休假制度。解决好拖欠职工工资和农民工工资问题。

　　（六）不断健全社会保障体系。完善城镇企业职工养老保险省级统筹，规范企业职工基本养老保险个人账户。从今年起，再连续三年提高企业退休人员养老金水平。积极推进事业单位养老保险制度改革，探索建立农村养老保险制度。健全城乡医疗救助制度，将新农合参合标准由每人50元提高到100元。逐步将高风险行业农民工纳入工伤保险覆盖范围。继续提高城乡低保标准，逐步将农村符合条件的贫困人口纳入低保范围。大力发展社会福利和慈善事业。改进和规范经济适用房制度，着力解决城市低收入家庭住房问题。搞好国有煤矿棚户区改造，确保完成改造任务。加强“菜篮子”产品市场监管，保持生产稳定、价格稳定和市场稳定。

　　（七）切实加强社会管理。健全应急管理体系，完善有关预案和制度，建立应急服务平台，提高危机管理和抗风险能力。落实安全生产责任制，遏制重特大事故发生。建立健全群众利益协调机制和社会矛盾疏导化解机制，着力从源头上预防和化解矛盾。深化城市社区建设，做好第六届村民委员会换届选举工作。加强社会组织的培育和管理，积极发挥各类社会组织的作用。进一步强化社会治安综合治理，完善社会治安防控体系，建设平安三门峡。加强食品药品安全监管，不断提高食品药品安全质量。落实计划生育基本国策，稳定低生育水平，优化人口结构，提高人口素质。加强法律援助，依法保障公民特别是妇女、未成年人和残疾人的合法权益。

　　深入开展国防教育，支持驻军和武警部队建设，抓好民兵预备役和人民防空工作。积极开展双拥共建活动，巩固和发展军政、军民关系。全面贯彻党的民族宗教政策，发挥宗教界人士和信教群众在促进经济社会发展中的积极作用。充分发挥工会、共青团、妇联等人民团体的桥梁纽带作用。进一步加强外侨、对台工作。搞好第二次经济普查，继续做好气象、史志、防震减灾等工作。

六、走生态良好的文明发展道路

　　（一）突出抓好节能节约。强化工作责任。认真落实节能减排统计、监测、考核实施方案和办法，严格落实节能减排责任，实行一票否决，打好节能减排攻坚战。淘汰落后产能。综合采取多种措施，逐步淘汰落后技术工艺，下决心关停一批能耗高、效益差、污染严重的小火电、小水泥、小刚玉砂等企业。加强节能管理。突出抓好年耗能5000吨标煤以上企业节能工作，加快高耗能企业的技术改造。强化企业内部管理，建设资源节约和环境友好型企业。严格新上项目准入标准，杜绝高耗能、高污染项目。更加重视建筑业、交通运输业、公用事业、政府机关和居民生活等领域的节能降耗，在全社会形成文明、节约的消费方式。搞好资源集约利用。严守基本农田红线，减少企业非生产性用地，引导企业积极利用荒丘、荒山建设项目。继续开展土地“三项整治”，加大对闲置和低效利用土地的清查处置力度，盘活存量土地。巩固禁用实心黏土砖成果，积极推广新型墙体材料，大力推广多层标准厂房。进一步优化煤炭、铝矾土、黄金资源配置，加快其他重要矿产资源整合。

　　（二）加强污染排放整治。继续抓好重点流域、区域和行业的环境污染综合治理，深入开展违法排污企业专项整治，做到新污染企业坚决不上，老污染企业坚决改造，改造不达标的企业坚决关停。下大力气治理大气污染，加强燃煤电厂除尘脱硫设施建设和运行监管，各类燃煤机组要全部完成脱硫设施建设并达标稳定运行。切实抓好污水处理厂的运行管理，加大污水处理费征收力度，加快建设污水处理配套管网。实施农村小康环保计划，治理农村面源污染，推广高效、低毒、低残留农药和测土施肥，搞好秸秆综合利用，加强规模化畜禽养殖污染治理，改善农村环境质量。

　　（三）大力发展循环经济。全面落实《循环经济发展实施方案》，逐步建立循环经济评价指标体系，稳步推进煤炭、电力、食品、化工等行业及农、林、牧等领域的循环经济发展。积极推广水泥行业低温余热发电、城市中水回用等先进技术。着力培育一批再生资源循环利用企业，大力推进废旧资源再生利用。

　　（四）全面建设生态三门峡。推进林业生态县、乡、村三级联创，建设一批重点林业生态工程，加快生态三门峡建设步伐。拓宽融资渠道，加大林业生态建设投入。继续实施天然林保护、重点地区防护林、速生丰产用材林基地等重点生态工程，确保完成造林41.17万亩，森林抚育和改造24.56万亩。继续抓好以天然林保护工程为主的资源保护和林业产业发展，林业总产值达到37.55亿元。

七、进一步扩大改革开放

　　（一）继续推进体制改革。进一步深化国有企业改革。巩固企业改革成果，完善股权结构，实现投资主体多元化。深入推进商贸、文化、建筑、粮食等非工业企业改革。全面推进集体林权制度改革。加快集体林地和宜林地产权、确权发证工作，实现“山有其主、主有其权、权有其责、责有其利”，形成林业良性发展机制。积极推动财政、金融改革和发展。深化财税改革，做好统一内外资企业所得税工作，依法加强税收征管；实施“民生财政”，优化支出结构，较大幅度增加社会保障、住房保障、卫生、教育等方面的支出。积极支持金融机构改革，加快城市信用社组建商业银行步伐。防范化解金融风险，不断优化金融生态环境。深化投资体制改革，继续推进交通、建设、水利等领域的改革。切实加强自主创新能力建设。深入实施《三门峡市中长期科学技术发展规划纲要》，围绕支柱产业发展，启动重大科技专项和重大科技工程。深化科技体制改革，优化资源配置，加快企业研发中心建设，促进产学研结合。鼓励发明创造，保护知识产权。

　　（二）加快发展开放型经济。全方位提高招商引资档次和质量。进一步搭建好对外开放和招商引资平台，强化措施，增强实效。办好第十四届三门峡国际黄河旅游节暨投资贸易洽谈会，加大对签约项目的跟踪落实力度，提高合同履约率、资金到位率和项目开工率。优化进出口商品结构。扩大先进装备和紧缺原材料的进口，着力培育新的出口品种，提高进出口效益。大力发展非公有制经济。进一步完善政策措施，鼓励企业加快技术创新，促进非公有制经济快速健康发展。优化产业结构，提高非公有制经济的产品档次和规模，力争营业收入超亿元的企业达到30个。完善中小企业信用担保体系，努力解决中小企业融资难问题。

　　（三）优化经济发展环境。综合运用行政、法律、经济和舆论等手段，优化政策环境、法治环境、服务环境、人文环境和信用环境，着力建设优化环境的长效机制。完善外商投资无偿代理制和便利化措施，加大企业周边和重点项目建设环境治理，营造亲商、安商、惠商的良好投资环境。强化市场流通、经营环境等综合治理，整顿和规范市场经济秩序。

八、加强服务型政府建设

　　（一）坚持依法行政。政府工作要努力实现决策民主化、管理法制化、工作高效化。自觉接受市人大及其常委会的法律监督、工作监督和市政协的民主监督，严格执行市人大及其常委会的决议、决定，广泛听取各民主党派、工商联和无党派人士的意见和建议。进一步加强政府法制建设，推进行政执法责任制，强化行政执法监督，规范行政权力。搞好行政复议，加强规范性文件的备案审查工作。强化政府层级监督，充分发挥监察、审计等专门监督的作用。进一步加强行政服务中心建设，减少行政审批事项。规范财政转移支付、土地和矿产资源、政府采购、国有资产转让等公共资源管理权力，建立健全决策权、执行权、监督权相互制约、相互协调的运行机制。

　　（二）推进管理创新。深化行政管理体制改革，转变职能、理顺关系、优化结构、提高效能，形成权责一致、分工合理、决策科学、执行顺畅、监督有力的行政管理体制。继续推进政企分开、政资分开、政事分开、政府与市场中介组织分开，充分发挥市场在资源配置中的基础性作用。进一步健全部门之间的协调配合机制，着力解决权责脱节、政出多门等问题，提高行政效能。

　　（三）完善公共服务。健全政府职责体系，更加注重履行社会管理和公共服务职能，积极改善民生，加强社会建设。完善公共服务体系，优化公共资源配置。全面推进政府信息公开，加强电子政务和政府门户网站建设，为人民群众提供优质便捷服务。

　　（四）转变工作作风。坚持求真务实、真抓实干，积极进取，昂扬向上，认真负责地做好各项工作，切实提高政府的公信力、执行力。切实改进会风、文风，严格控制和规范各种检查、评比、达标活动。建立健全符合科学发展的政府工作考核体系，确保责任目标落实到位。进一步提高公务员管理的法制化、规范化水平，努力建设一支政治坚定、业务精通、清正廉洁、作风优良的公务员队伍。

　　（五）建设廉洁政府。严格落实党风廉政建设责任制，不断健全教育、制度、监督并重的惩治和预防腐败体系。牢记“两个务必”，厉行节约，勤俭办事，自觉抵制各种不良风气的侵蚀，坚决反对铺张浪费和奢靡之风。加大专项治理力度，重点解决土地征用、房屋拆迁、环境保护、食品药品安全等群众反映强烈的问题，坚决纠正损害群众利益的不正之风，以反腐倡廉的实际成果取信于民。

　　各位代表！今年是全面贯彻党的十七大精神的关键之年，做好政府工作，责任重大，使命光荣。让我们在省委、省政府和市委的正确领导下，高举中国特色社会主义伟大旗帜，团结拼搏，开拓进取，为早日实现再造一个三门峡、跻身中原崛起第一梯队的宏伟目标而努力奋斗！
